# Evaluating the Efficacy of Omalizumab in Severe Cedar Seasonal Allergic Rhinitis in Japan

**DOI:** 10.7759/cureus.63714

**Published:** 2024-07-02

**Authors:** Takakazu Goto, Toru Miwa, Kousuke Hashimoto, Kazuki Amesara, Yuko Unno, Hirokazu Sakamoto

**Affiliations:** 1 Otolaryngology, Minamiosaka Hospital, Osaka, JPN; 2 Otolaryngology, Osaka Metropolitan University, Osaka, JPN; 3 Otolaryngology, Kytoto University, Kyoto, JPN; 4 Otolaryngology, Osaka City Juso Hospital, Osaka, JPN; 5 Otolaryngology, ISEIKAI International General Hospital, Osaka, JPN

**Keywords:** sublingual immunotherapy, allergic rhinitis, seasonal allergy, cedar allergy, omalizumab

## Abstract

Background: Traditional treatments for cedar seasonal allergic rhinitis include second-generation antihistamines, nasal corticosteroids, and sublingual immunotherapy (SLIT). Omalizumab (Xolair®), an anti-immunoglobulin E (IgE) monoclonal antibody, is an additional option for severe cases unresponsive to existing therapies. Numerous studies have demonstrated the therapeutic effectiveness of omalizumab for cedar seasonal allergic rhinitis; however, most reported results after only up to four weeks of follow-up. Therefore, this study evaluates the clinical efficacy of omalizumab throughout one whole cedar pollen season. Subjects and methods: This study included patients from our department and the Otorhinolaryngology Department of Minami Osaka Hospital between 2021 and 2023 who were ≥ 12 years old and had serum total IgE levels of 30-1,500 IU/mL, a baseline weight of 30-150 kg, and persistent severe nasal symptoms despite conventional treatments. Patients taking oral steroids at the time of enrollment or had fewer than two omalizumab doses were excluded. Forty-six patients (26 males, 20 females; mean age, 19.1 ± 11.2 years) met these criteria and received subcutaneous omalizumab every 2 or 4 weeks based on their IgE levels and weight. Symptoms were assessed at baseline and 4, 8, and 12 weeks post-administration using the Total Nasal Symptom Score (TNSS) and the Japanese Standard Quality of Life Questionnaire (JRQLQ No. 1) for allergic rhinitis. Results: Thirty-six patients were followed up for 8 weeks and 13 for 12 weeks. TNSS significantly improved from 6.6 to 4.5 at 4 weeks, 4.2 at 8 weeks, and 4.1 at 12 weeks (p<0.05). Nasal discharge, sneezing, nasal obstruction, itchy eyes, and tearfulness showed significant improvements (p<0.05). Quality of life scores improved in daily activities, sleep, and physical health from week 4 to week 12. Discussion: Consistent with previous findings, omalizumab significantly improved nasal and ocular symptoms and quality of life in patients with severe cedar seasonal allergic rhinitis. Despite many patients discontinuing the drug after eight weeks due to high costs, the drug's effectiveness in preventing symptom recurrence suggests potential long-term benefits. Combining omalizumab with SLIT showed no significant differences in outcomes; however, further pharmacoeconomic studies are warranted to evaluate cost-effectiveness. Conclusion: Omalizumab proved to be an effective treatment for severe cedar seasonal allergic rhinitis, providing significant symptom relief and quality of life improvements. Further studies should investigate its long-term efficacy and safety, including potential adverse effects and the development of anti-omalizumab antibodies.

## Introduction

The prevalence of cedar seasonal allergic rhinitis has grown considerably in recent years, rising from 16.2% in 1998 to 38.8% in 2019 [1]. Treatment options include oral second-generation antihistamines, nasal corticosteroids, and sublingual immunotherapy [2]. In December 2019, the use of omalizumab (Xolair®), a humanized anti-human immunoglobulin E (IgE) monoclonal antibody, was approved for patients with severe disease who demonstrated inadequate response to existing therapies [3]. Omalizumab functions by inhibiting the binding of IgE to high-affinity IgE receptors (FcεRI) located on the cell membranes of basophils and mast cells, consequently preventing the release of inflammatory mediators, such as histamine, through the degranulation of these cells, thereby blocking type I allergic reactions.

Although numerous studies have been published in Japan and worldwide on the therapeutic effects of omalizumab for cedar seasonal allergic rhinitis [4-7], most of these studies reported results after only up to 4 weeks of follow-up, and the sample sizes were often limited.

In this study, we investigated the clinical efficacy of omalizumab during the cedar pollen dispersal period over one whole season of cedar seasonal allergic rhinitis.

## Materials and methods

For this study, we included patients who visited our department of otolaryngology in Japan between 2021 and 2023 and met the following criteria: age≥ 12 years; serum total IgE level, 30-1500 IU/mL; baseline weight, 30-150 kg; presence of persistent severe nasal symptoms despite treatment with histamine H1 receptor antagonists, nasal corticosteroids, and ophthalmic antihistamines; and willingness to receive omalizumab. We excluded patients who were taking oral steroids at the time of enrollment and had a receipt of fewer than two doses of omalizumab. Of the 48 patients who requested dosing, 46 met the inclusion criteria and were enrolled in the study. Excluded two patients who received omalizumab only once. Patients received subcutaneous omalizumab every 2 or 4 weeks based on their serum total IgE levels and baseline body weight, with dosing schedules consistent with those used for asthma patients. The following parameters were quantified before administration: serum nonspecific IgE levels, serum cedar-specific IgE antibody titers (measured using fluorimetric enzyme-linked immunoassay (FEIA), Thermo Fisher Diagnostics K.K., Japan), omalizumab dose, administration interval, and the presence or absence of concomitant sublingual immunotherapy (SLIT). Allergic rhinitis nasal and ocular symptoms, such as runny nose, sneezing, nasal congestion, and itchy eyes, were assessed at baseline and 4, 8, and 12 weeks after the initial omalizumab administration, using the Total Nasal Symptom Score (TNSS) and the Japanese Standard Quality of Life (QOL) Questionnaire for Allergic Rhinitis (JRQLQ No. 1) [8]. Additionally, QOL was evaluated using a subjective nasal symptom questionnaire, comprising 17 questions divided into six domains: usual activities; outdoor activities; social functioning; sleep problems; general, physical and emotional functioning; and overall face score. Patients who chose not to receive the second or subsequent doses were examined and surveyed at a follow-up visit approximately one month later. Statistical analyses were performed using analysis of variance (ANOVA) with Tukey's post hoc test, utilizing GraphPad Prism software (version 10.0.0, CA, USA). A p-value of < 0.05 was considered statistically significant. This study was conducted with the approval of the Ethics Review Committee of Osaka Metropolitan University (No. 2020-174). All patients and their parents, if they were under 20 years of age and wanted to participate in this study, had their informed consent. This study was performed according to the Guidelines for Strengthening the Reporting of Observational Studies in Epidemiology (STROBE).

## Results

The baseline characteristics of the patients are presented (Table 1). With regard to pre-administration treatment, there were many cases of poor medication adherence and compliance, but we did not tabulate the percentage of these cases. Thirty-three patients were followed up for 8 weeks, and 13 were followed up for 12 weeks. All patients who opted out of the study reported improved subjective nasal symptoms and discontinued omalizumab treatment.

**Table 1:** Patient background SD: standard deviation; SLIT: sublingual immunotherapy

Variable	N = 46
Sex: male:female	26:20
Age: years (mean ± SD, range)	19.1 ± 11.2 (12–79)
Serum total IgE levels at baseline: IU/mL (mean ± SD)	606.8 ± 518.1
Specific IgE levels against cedar pollens: IU/mL (mean ± SD)	4.5 ± 1.2
Specific IgE levels against house dust: IU/mL (mean ± SD)	3.4 ± 2.2
Total dose of Omalizumab: mg (mean ± SD)	412.2 ± 130.0
Dosage Interval: 2:4 weeks	20:26
Patients on anti-allergic drugs	8
Patients on nasal spray steroids	7
Patients on SLIT against cedar pollens	20
Patients on SLIT against mite	18

**Figure 1:**
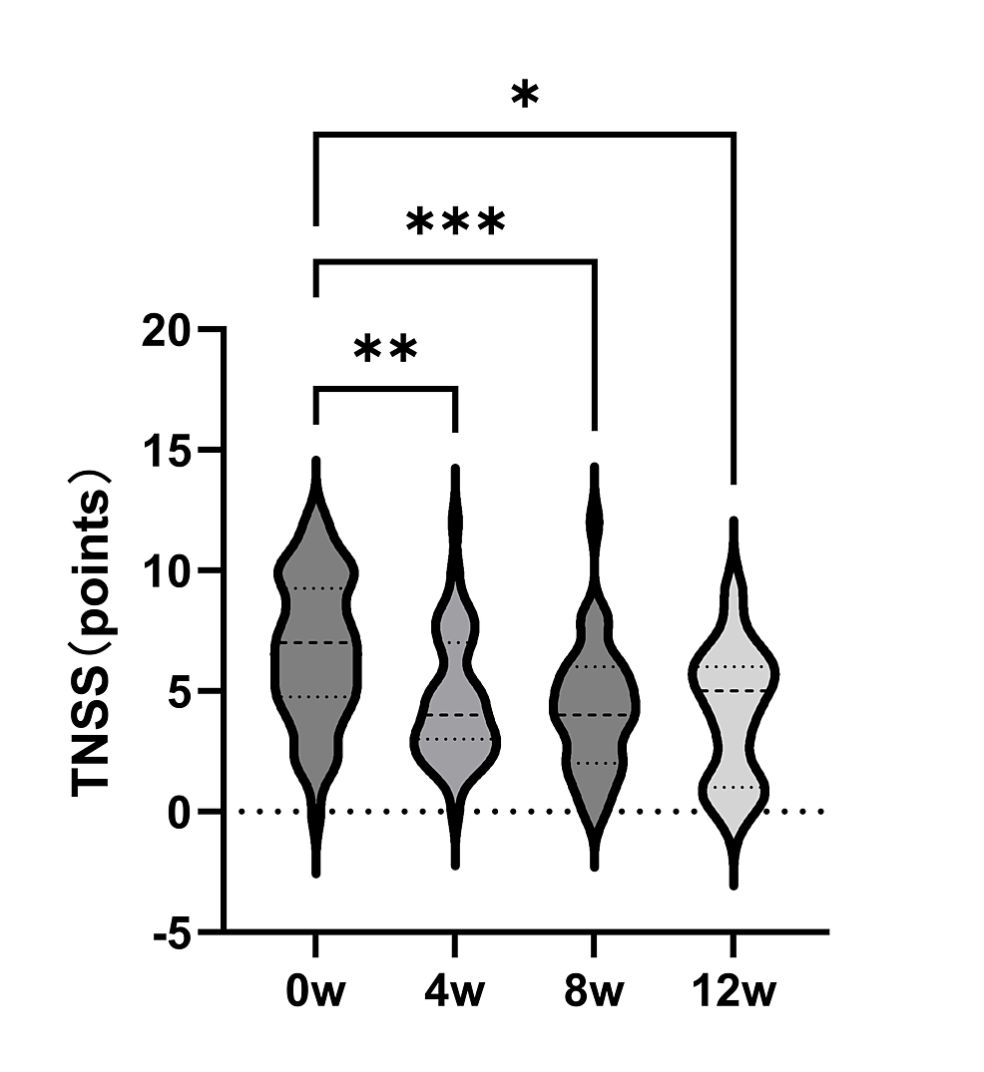
Total nasal symptom score The patient's symptoms were significantly improved after four, eight, and 12 weeks of treatment TNSS: total nasal symptom score *: p < 0.05; **: p < 0.01; ***: p < 0.001

The average TNSS (the lower, the better) significantly improved from 6.6 before treatment to 4.5 after 4 weeks (p = 0.002), 4.2 after 8 weeks (p < 0.001), and 4.1 after 12 weeks (p = 0.02; Figure 1).

**Figure 2:**
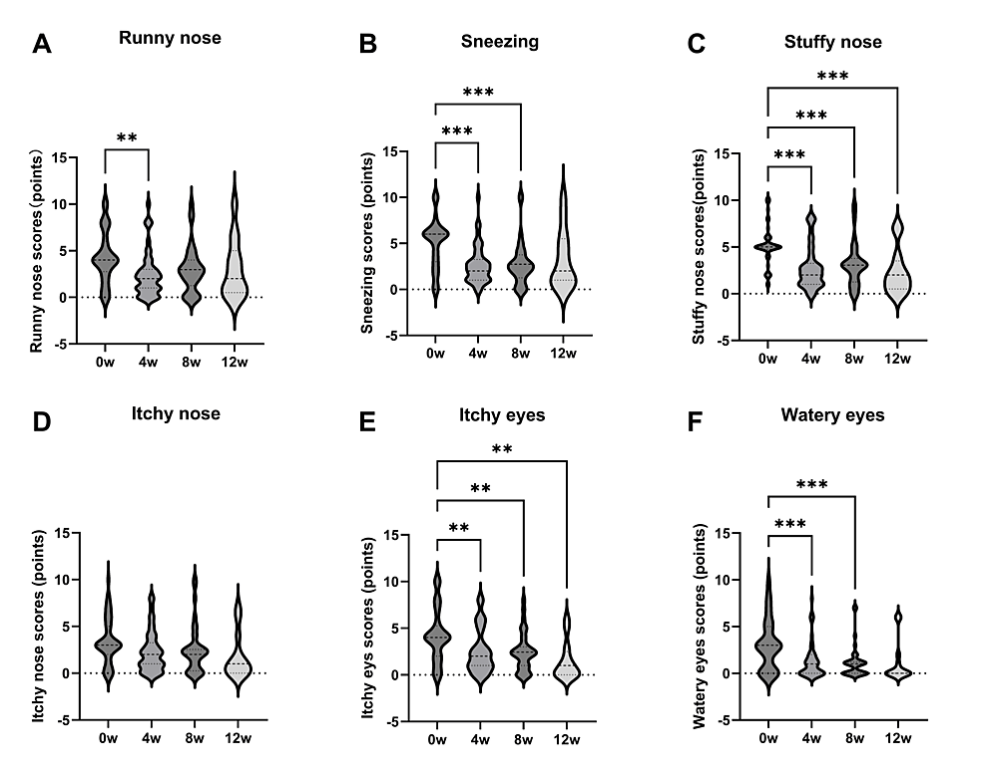
Japanese Standard Quality of Life Questionnaire (JRQLQ) nasal symptom scores Nasal discharge was significantly improved after four weeks of treatment (A). Sneezing was significantly improved at weeks four and eight (B). Nasal obstruction and itchy eyes improved with time (weeks 4-12; C, E). Teary eyes also showed improvement over time (F) at weeks four and eight. No significant change was observed in nasal itching (D). **: p < 0.01, ***: p < 0.001

JRQLQ scores (the lower, the better) were recorded for nasal and ocular symptoms; nasal discharge improved significantly from 4.2 before treatment to 2.4 at 4 weeks post-treatment (p = 0.006; Figure 2A). Sneezing also improved significantly from 2.6 before treatment to 2.3 and 2.2 at 4 and 8 weeks, respectively (p < 0.001 for both; Figure 2B). Additionally, nasal obstruction and itchy eyes showed improvement over time; nasal obstruction improved from 5.0 before treatment to 2.7, 3.1, and 2.3 at 4, 8, and 12 weeks, respectively, while itchy eyes improved from 3.4 before treatment to 2.3, 2.3, and 1.7 at 4, 8, and 12 weeks, respectively (Figure 2C, 2E). Tearfulness improved from 3.0 before treatment to 1.3 and 1.1 at 4 and 8 weeks, respectively (p < 0.001 for both; Figure 2F). However, there was no significant change in nasal itching (Figure 2D).

Additional JRQLQ scores reflected QOL improvements in domains related to daily life, sleep, and physical health from 4 to 12 weeks after initiating treatment. Daily life improved from 1.3 before administration to 0.6, 0.5, and 0.2 at 4, 8, and 12 weeks, respectively. Sleep improved from 1.5 before administration to 0.8, 0.5, and 0.4 at 4, 8, and 12 weeks, respectively. As for physical health, the score improved from 1.5 before administration to 0.8, 0.5, and 0.3 at 4, 8, and 12 weeks, respectively.

**Figure 3:**
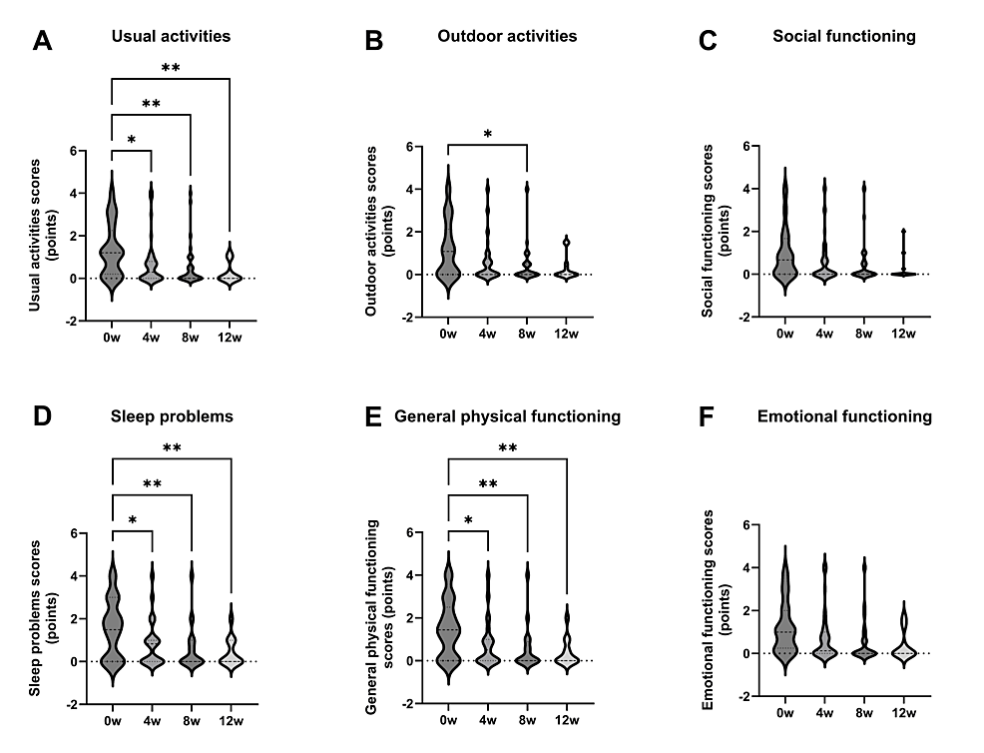
Japanese Standard Quality of Life Questionnaire (JRQLQ) quality of life scores Daily life, sleep, and physical health domains improved over time from four to 12 weeks after treatment (A, D, E). Outdoor activities showed improvement at week eight (B). No significant changes were observed in social life or mental life domains (C, F). *: p < 0.05, **: p < 0.01

Improvement was also observed in outdoor activities at 8 weeks (from 1.2 before administration to 0.4 after 8 weeks). However, no significant changes were observed in social life or mental health (Figure 3A-3F).

**Figure 4:**
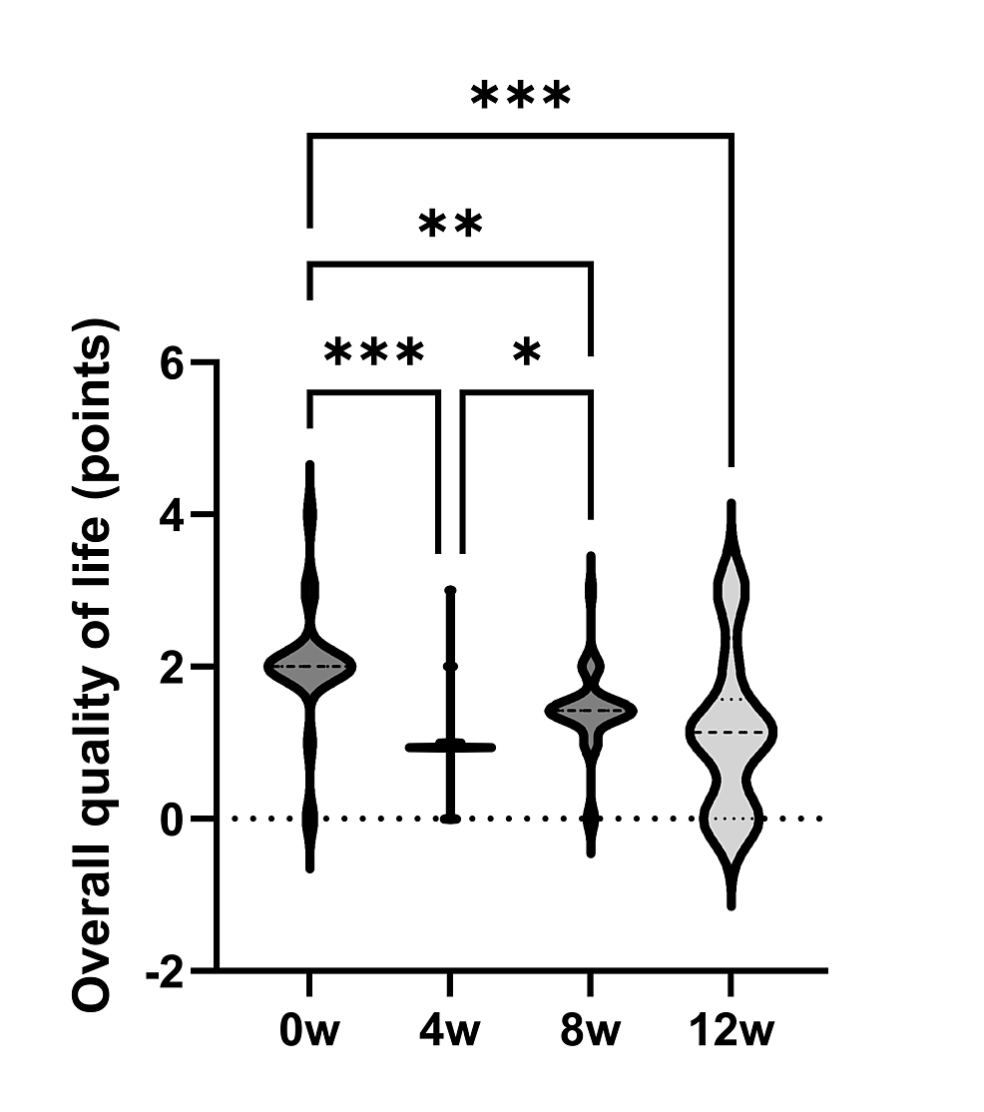
Japanese Standard Quality of Life Questionnaire (JRQLQ) overall face score Improvement over time was observed from four to 12 weeks after treatment, although a transient deterioration was observed between weeks four and eight. *: p < 0.05, **: p < 0.01, ***: p < 0.001

Lastly, the JRQLQ overall face score summary showed improvement from 2.0 before treatment to 0.9, 1.4, and 1.1 after 4, 8, and 12 weeks, respectively (Figure 4).

**Figure 5:**
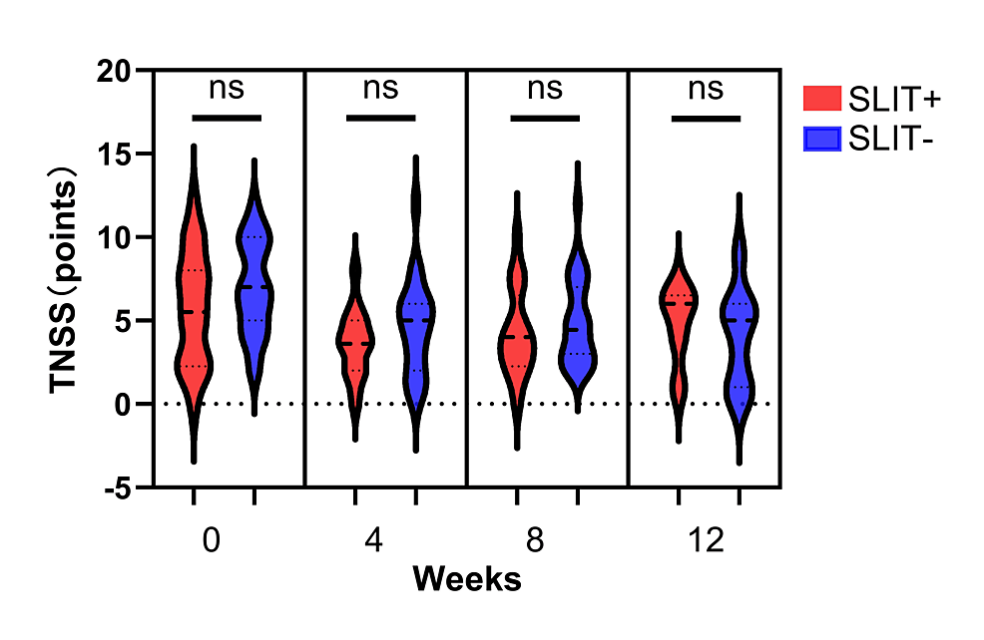
Comparison between TNSS in patients with and without SLIT No significant difference was observed between the two groups at any point of time. TNSS: total nasal symptom score; SLIT: sublingual immunotherapy; ns: non-significant difference. ns: not significant.

**Figure 6:**
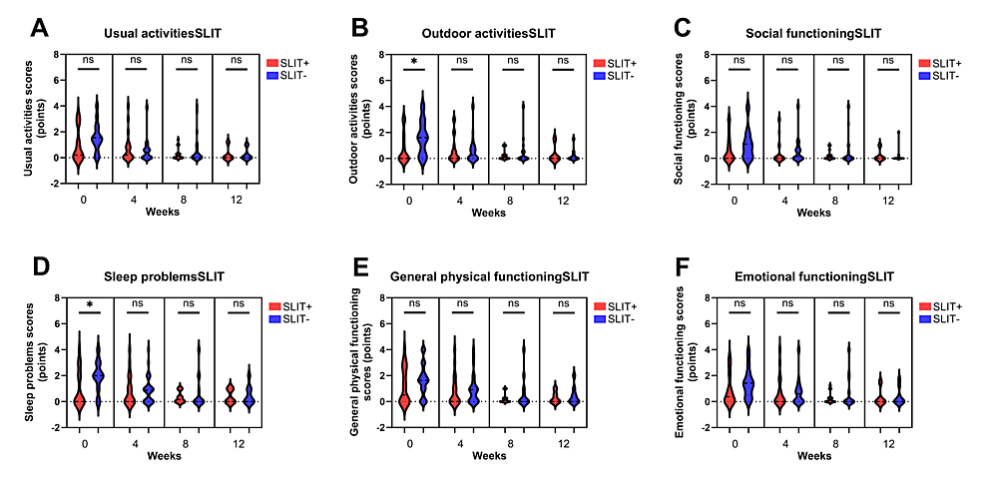
Comparison between the Japanese Standard Quality of Life Questionnaire (JRQLQ) scores in patients with and without SLIT. Outdoor activities and sleep problem scores were significantly higher before omalizumab initiation without SLIT than with SLIT (B, D). Other domains showed no significant differences throughout the course of the study (A, C, E, F) TNSS: total nasal symptom score; SLIT: sublingual immunotherapy; ns: non-significant difference. *: p < 0.05, ns: not significant.

We compared TNSS between patients with and without SLIT for cedar allergy, no significant differences were observed at any time point (Figure 5). Nevertheless, patients without SLIT had significantly worse sleep problems before omalizumab initiation than those receiving SLIT; however, no notable differences were observed throughout the rest of the treatment course (Figure 6).

## Discussion

Nasal symptoms of allergic rhinitis significantly impair patients’ QOL, and the sedative effects of drug treatment have been shown to impair learning and work efficiency [9,10]. With a prevalence exceeding 30%, its societal impact is substantial. Our results demonstrated that omalizumab treatment for cedar seasonal allergic rhinitis improves nasal and ocular symptoms, various QOL aspects, and overall status from the fourth week after treatment initiation, with sustained improvements through week 12. These findings are consistent with previous reports [3,4] and support the notion of omalizumab’s long-term efficacy throughout one cedar season.

The safety of omalizumab has been well-documented in treating bronchial asthma [11], suggesting its potential for seasonal use during the cedar pollen season. However, many patients discontinued the drug after eight weeks of treatment; while this may be attributed to the efficacy of the drug, which prevented recurrence, the high cost likely also discouraged continued use. Pharmacoeconomic studies are warranted to investigate this issue. Nonetheless, previous research has shown a 75% reduction in days missed from work or school in patients treated with omalizumab compared with those on placebo treatment [6], indicating that pharmacoeconomic analysis might further support the use of omalizumab. In addition, our results indicate that mandatory omalizumab administration might be a good option for patients with poor daily medication compliance.

Combining omalizumab with immunotherapy for allergic rhinitis has been reported to enhance efficacy [12]. In this study, we observed 20 cases of combination with SLIT. SLIT is reportedly associated with high patient satisfaction rates-85% at the end of one season and 92.4% at the end of two seasons and is known to significantly improve symptoms, including other allergic conditions [13]. Allergen immunotherapy, such as SLIT, is a fundamental treatment for allergic rhinitis. Proper administration can lead to symptom improvement over time, and continuing treatment for more than three years can maintain efficacy long after discontinuation. However, our results indicate that severe allergic symptoms can occur during the cedar pollen dispersal period even with SLIT, and omalizumab is effective in such cases as well as in patients not on SLIT.

While omalizumab is primarily used for refractory bronchial asthma, its long-term safety remains a concern. Reports of anti-omalizumab antibodies have been detected in patients with other diseases treated with omalizumab [14], highlighting the need for further investigation into omalizumab’s long-term efficacy and safety through employing larger study populations.

This study faced several limitations that should be addressed in future research. First, patient evaluation was based on nasal symptom scores and other questionnaires and did not include scoring of intranasal findings or measurement of IgE levels during the study period. Second, the study could not exclude the placebo effect of omalizumab injections. Placebo effects are not uncommon in allergic rhinitis studies; therapeutic interventions with oral or topical medications typically produce a placebo effect of approximately 30% to 40% [15]. Third, the adverse events of omalizumab were not investigated. Although no major adverse events were observed, further detailed investigation of the safety profile of omalizumab is warranted. Fourth, since cedar pollen dispersal usually lasts two to three months, the cedar pollen dispersal is over by week 12, and the improvement at week 12 might be due to natural improvement as well as the effect of treatment. Fifth, this study's lack of a control group is a limitation.

## Conclusions

The study explored the clinical efficacy of omalizumab in treating cedar seasonal allergic rhinitis over one entire pollen season. The treatment significantly improved nasal and ocular symptoms, enhancing patients’ QOL. Specifically, omalizumab showed substantial improvements in symptoms such as nasal discharge, sneezing, nasal obstruction, and itchy eyes from as early as four weeks into the treatment, and these improvements were sustained through 12 weeks. Omalizumab was also effective in patients with severe allergic symptoms despite receiving SLIT. This indicates that omalizumab can be a valuable option for patients with inadequate responses to traditional therapies. However, the high cost of omalizumab may limit its long-term use, as observed with the discontinuation of treatment by several patients after eight weeks. This underscores the need for pharmacoeconomic studies to understand better the cost-benefit ratio of omalizumab treatment for seasonal allergic rhinitis.

Furthermore, combining omalizumab with SLIT may enhance treatment efficacy, as suggested by the 20 cases observed in this study. Despite the positive outcomes, the study highlights the necessity for further research with larger sample sizes to explore the long-term efficacy and safety of omalizumab and investigate potential adverse effects and the impact of anti-omalizumab antibodies. Overall, this study reinforces the role of omalizumab as an effective treatment for cedar seasonal allergic rhinitis, providing significant symptom relief and improving patients’ QOL while identifying areas for future research to optimize its use.
